# Suicide Risk in Digestive Cancer Patients: A Systematic Review of Sociodemographic, Psychological, and Clinical Predictors

**DOI:** 10.3390/cancers17091427

**Published:** 2025-04-24

**Authors:** Diana Elena Lazar, Bianca Hanganu, Roxana Postolica, Camelia Liana Buhas, Cristian Paparau, Beatrice Gabriela Ioan

**Affiliations:** 1 Doctoral School, Grigore T. Popa University of Medicine and Pharmacy, 700115 Iasi, Romania; lazardianaelena@yahoo.com; 2 Department of Oncology, Municipal Hospital “St. Hierarch Dr. Luca”, 601048 Onesti, Romania; 3 III-rd Medical Department, Legal Medicine, Faculty of Medicine, Grigore T. Popa University of Medicine and Pharmacy, 700115 Iasi, Romania; beatrice.ioan@umfiasi.ro; 4 Department of Psychology, Regional Institute of Oncology, 700483 Iasi, Romania; roxana.postolica@yahoo.com; 5 Department of Morphological Disciplines, Faculty of Medicine and Pharmacy, University of Oradea, 410087 Oradea, Romania; cameliabuhas@yahoo.com; 6 Dambovita County Forensic Medicine Service, Targoviste Emergency County Hospital, 130086 Targoviste, Romania; cristianpaparau@yahoo.com

**Keywords:** suicide, digestive system cancer, risk factors, death, dying

## Abstract

A cancer diagnosis often triggers profound psychological and emotional distress as individuals reflect on existential issues such as life and death. The aim of this review was to provide estimates of suicide risk associated with digestive cancer worldwide, and to identify sociodemographic, psychological, and clinical factors associated with suicide risk in patients with digestive cancer. Materials and methods: The literature search was guided by the following question: What is the relationship between suicide and digestive cancer, and what sociodemographic, psychological, and clinical factors contribute to the risk of suicide in these patients? We searched PubMed, PsycINFO, Embase, CINAHL, and Web of Science, and systematically reviewed the evidence, according to PRISMA guidelines, from relevant articles on the association between digestive system cancers and suicide outcomes published over a 14-year period (2011–2024). Eligible studies were searched in the main scientific databases up to August 2024. Results: There are many reasons why people die by suicide, including challenges faced by patients in mentally adapting to their new condition and physical illness. Studies have shown that the highest suicide rates in digestive cancer patients are observed in males, older age groups, single people, those with a poor cancer prognosis, and those with a lack of treatment provision (surgery or chemotherapy). The risk of suicide peaks at six months post-discrimination, remains stable for three years, and then declines. Conclusions: Systematic changes in cancer care, such as aggressive treatment of pain and physical symptoms, management of delirium and cognitive impairment, routine screening, increased monitoring, and proactive measures for high-risk patients, can play a critical role in preventing unnecessary deaths and addressing the increased vulnerability of cancer patients, underscoring the need for targeted psychological support and early intervention, especially during critical periods like diagnosis and post-treatment recovery.

## Simple Summary

There are many reasons why people commit suicide, including the challenges faced by patients with digestive cancer in mentally adapting to their new conditions and physical illnesses. It is often the result of a complex interplay of risk and protective factors at individual, interpersonal, community, and societal levels. However, the exact reasons behind the suicide rate remain a matter of investigation. To prevent suicide in this population, we need to maximize protective factors by increasing mental adaptability to the cancer diagnosis, ensuring early palliative care, and regularly screening cancer patients for distress and suicide risk, especially during periods identified as high risk.

## 1. Introduction

Understanding the global cancer burden is critical to ensuring that everyone has the opportunity to prevent, detect, treat, and survive cancer [1]. The American Cancer Society released a Global Cancer Statistics report in 2024, which estimates that approximately 20 million new cases of cancer were diagnosed in 2022, and approximately 9.7 million individuals died from cancer during that time. Moreover, the number of global cancer cases is predicted to reach 35 million by 2050 [2,3]. Additional findings from the report demonstrate that approximately one in five individuals develop cancer in their lifetime, with approximately one in nine men and one in twelve women dying from the disease. The most frequently diagnosed cancer in 2022 was lung cancer, with 2.5 million new cases accounting for 12.4% of all new cancer diagnoses in 2022. Other commonly diagnosed cancers in 2022 included female breast cancer (11.6%), colorectal cancer (9.6%), prostate cancer (7.3%), and stomach cancer (4.9%). The leading cancers contributing to the 2022 cancer mortality rate included lung lancer (18.7%), colorectal cancer (9.3%), liver cancer (7.8%), female breast cancer (6.9%), and stomach cancer (6.8%). [3]. The most recent year for which incidence and mortality data are available lags 2–3 years behind the current year because of the time required for data collection, compilation, quality control, and dissemination [4].

A person’s chances of survival depend on the type of digestive cancer: colorectal cancer (the most common cancer of the digestive tract) has the highest 5-year relative survival rate (69%) [5]. In comparison, pancreatic cancer (the second most common cancer of the digestive system) has the lowest 5-year relative survival rate of all specified cancers of the digestive system (8.7%) [5]. Nevertheless, the implications of a diagnosis that can be fatal and the path of cancer treatment and recovery continue to present significant and often ignored issues of vulnerability to psychological distress (chronic depression and anxiety) [6,7,8], reduced quality of life [9,10,11,12], disability and debilitation [13,14], and reduced engagement in health-promoting behaviors [15,16,17] at different points in the patient’s life course.

The definition of “suicide” in the Cambridge Dictionary [18] is “the act of killing yourself intentionally”. Suicide is described as a consequence of unresolved suffering [19], and represents a considerable health burden for society. Although few cancer patients die by suicide compared to the number who report suicidal ideation and a desire for hastened death, cancer patients are reported to have about twice the risk of suicide compared to the general population in the USA [20] and other medical populations, with recent studies putting this risk at four times that of the general population [21].

The comorbidity of suicide and major depression is widespread both in the general population and in cancer patients [22]. In a study of advanced cancer patients, the incidence of depression was found to be exceptionally high in this group; 58% of patients who reported a strong desire for hastened death also met the criteria for major depression. Depression was found to be a strong predictor of the wish to die [23]. Hopelessness was identified as an even stronger predictor than depression for suicidal ideation and the desire for hastened death in advanced cancer patients [24]. However, the co-existence of depression and hopelessness appears to be the most potent clinical marker for a high desire for hastened death and completed suicide [25].

“Demoralization syndrome”, which includes feelings of hopelessness, helplessness, incompetence, dependence, burden, loss of meaning, and existential distress, is also an indication of a higher risk of death wishes and suicide [26]. Several studies have shown that most cancer suicides are preceded by inadequately treated or poorly tolerated pain, and that pain significantly predicts suicide, a desire for death, and requests for euthanasia [21,26,27].

Psychiatric consultation data from three UK cancer centers showed that almost one-third of patients with cancer and comorbid major depression had the DSM symptom “thoughts of death or suicide” [28].

Since the main goals of supportive psychotherapy (SP) are to help patients alleviate emotional symptoms and resolve problems, regain balance and maintain stability, and achieve better adjustment, coping, and functioning, the use of SP in cancer treatment is extremely important. The priorities for cancer patients are to improve patients’ mental adaptation to their difficult situation by enhancing their sense of self-efficacy, confidence, and hope, allowing them to continue to engage with life, regulate their emotions, and alleviate the anxiety and confusion caused by the cancer trauma.

The aim of this review is to provide estimates of the suicide risk associated with digestive cancer worldwide, and to identify sociodemographic, psychological, and clinical factors associated with suicide risk in patients with digestive cancer, through drawing some conclusions based on research conducted over the last 14 years (2011–2024). By investigating the relationship between the location of the cancer and these dimensions, patterns can be identified that will enable targeted interventions and can improve patient care in oncology.

## 2. Materials and Methods

### 2.1. Data Sources and Search Strategy

**Figure 1:**
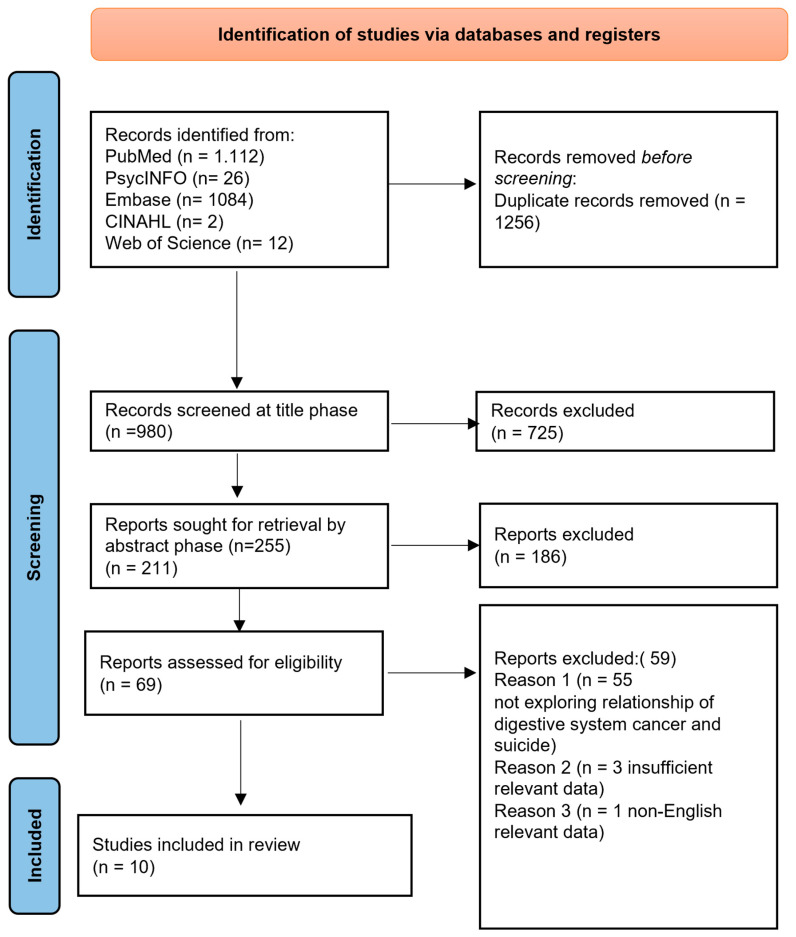
A PRISMA 2020 Flow Diagram illustrating the search procedure for selecting studies for consideration.

In conducting this systematic review, we followed the PRISMA (Preferred Reporting Items for Systematic Reviews and Meta-Analysis Guidelines) statement, which provides a rigorous process for conducting scientific work such as systematic reviews. It consists of a 27-item checklist and a four-phase flowchart (see Figure 1), designed to help authors improve their reporting of systematic reviews and meta-analyses. The results were limited to articles published in English during the last 14 years.

On 1 August 2024, we searched MEDLINE (through PubMed), PsycINFO, Embase, CINAHL, and Web of Science (Table 1), without restrictions on geographical location. In PubMed, we used the search term (“suicide” [Mesh] AND (“digestive cancer” [Mesh] OR “esophageal cancer” [Mesh] OR “gastric cancer” [Mesh] OR “pancreatic cancer” [Mesh] OR “colorectal cancer” [Mesh] OR “liver cancer”). In Embase, we used the search term (exp suicide) AND (digestive cancer system OR esophageal cancer OR gastric cancer OR pancreatic cancer OR colorectal cancer). In PsycINFO, we used the search term (exp suicide/OR “death” and “dying”) AND (exp “digestive cancer tract”/OR “esophageal cancer”/OR “gastric cancer”/OR “pancreatic cancer”/OR “colorectal cancer”). In CINAHL, we used the search term ((MH “suicide”) OR (MM “death and dying”)) AND ((MH “digestive system cancer”) OR (MM “esophageal cancer”) OR (MM “gastric cancer” OR “pancreas cancer”/OR “colorectal cancer”)) OR “liver cancer”. In Web of Science, we used the search term (TOPIC: (“suicide”) OR Title: (“death” and “dying”) AND (TOPIC: (“mortality”) OR TOPIC: (“digestive system cancer”) OR TOPIC: (“survival rate”) OR TOPIC: (“premature mortality”) OR TOPIC (“esophageal cancer”) OR TOPIC (“gastric cancer”) OR TOPIC (“pancreas cancer”)/OR TOPIC (“colorectal cancer”) OR TOPIC (“liver cancer”).

Articles included in the study had to be full-text research articles published in English that met the following inclusion criteria: (1) the population consisted of women and men with cancer of the digestive system; (2) the outcome variable included the assessment of suicide rate. The exclusion criteria included the following: (1) non-English articles; (2) the participants of the study were not digestive cancer patients (i.e., the study population had other types of cancer or other diseases); (3) systematic, literature, annual, and clinical reviews, books, unpublished articles, doctoral dissertations, commentaries, and meeting and conference abstracts and proceedings.

### 2.2. Data Collection Process

After introducing the above keywords and Boolean operators, as well as filters related to language in all databases, a total of 2236 articles were found. Finally, ten studies met the eligibility criteria and were included in the systematic review. Figure 1 shows a Flow Diagram providing an overview of the selection process, through which the data were systematically extracted.

## 3. Results

### 3.1. Findings

According to the search strategy, a total of 2236 articles were identified. However, 1256 records were duplicated. Therefore, of the total 980 articles, 911 were excluded after reading the titles and abstracts. As such, a total of 69 studies were selected based on the eligibility criteria. Of these, 59 were excluded, and finally, a total of 10 studies were selected to be included in the review.

### 3.2. Study Characteristics

The general characteristics of the studies listed in the review (*n* = 10) can be found in Table 2. Articles were published from 2011 to 2024 in a variety of scientific journals with different aims and scopes.

#### Participant Characteristics and Variables

Sociocultural and personal characteristics of participants are key factors to be taken into account when examining different studies. In the studies included in this review, suicide rates were calculated according to the number of suicides that were reported per 100,000 person-years of follow-up time, while standardized mortality ratios (SMRs) were calculated using the ratio between observed suicides and expected suicides.

Study 1 [29] included 199,604 participants from the National Cancer Institute’s Surveillance, Epidemiology, and End Results (SEER) program who were diagnosed with pancreatic cancer between 2000 and 2018. Their sex, race, state registered in the database, age at diagnosis, year of diagnosis, marital status, primary tumor location in the pancreas (head, body, tail, or other), histologic type, histologic grade, SEER summary stage (localized, regional, distant), presence of radiotherapy records, evidence of chemotherapy records, history of cancer-related surgical procedures, and survival months after diagnosis were recorded as patient characteristics of interest. Patients whose age, race, follow-up duration, or diagnosis at autopsy were unknown were not included in the study cohort.

Study 2 [30] investigated the suicide rate in patients with liver cancer using the primary localization codes for the liver and the morphologic codes based on the International Classification of Diseases for Oncology (3rd edition) codes for HCC. A total of 77,630 men and 24,937 women with HCC diagnosed between 1975 and 2016 were included in the study. The software SEER-Stat (version 8.3.6) was used to search for the following variables: (1) year of diagnosis; (2) sex; (3) age at diagnosis; (4) marital status; (5) purchased/referred care delivery area (PRCDA) region; (6) histologic grade; (7) stage according to SEER; (8) surgical intervention; (9) radiation therapy; (10) chemotherapy; (11) months of survival; and (12) race. In addition, the following cases were excluded: (1) those with a lack of information regarding age or race; (2) those with a lack of active follow-up; (3) cases where an autopsy was performed or where a death certificate was issued.

In Study 3 [31], patients who had esophageal cancer were selected from the Surveillance, Epidemiology, and End Results Repository from 1975 to 2016. The following cases were excluded: (1) autopsy or death certificate cases; (2) cases with non-positive histology; (3) cases with unknown follow-up; (4) cases with unknown age; or (5) cases with unknown race.

Study 4 [32] aimed to answer the question, “What is the risk of suicide following a cancer diagnosis in England, and which subgroups of patients are at particular risk?”, in a population-based study consisting of 4,722,099 adult patients (aged between 18 and 99 years) having received a cancer diagnosis between 1 January 1995 and 31 December 2015. The source of information was the Public Health England National Cancer Registry Database. All patients included in the study were inhabitants of England when the diagnosis was made. Patients who had cancer as the only diagnosis on death certificate were excluded, as they were unaware of their cancer. For individuals who died in the same day as diagnosis, the risk period consisted of 1 day. To determine the cause of death, the Office for National Statistics was traced.

Study 5 [33] included all patients with tumors of the digestive tract, as defined by the SEER site recode ICD-O-3/WHO 2008, diagnosed between 2000 and 2014. Variables available in the SEER database included patient age at diagnosis, cancer type, stage, year of diagnosis, surgery, demographic characteristics (sex, race, and marital status), and survival time calculated from the date of diagnosis.

Study 6 [34] evaluated a number of 20,765 patients diagnosed with primary colorectal cancer, covering the period between 1998 and 2012 and found in the Lithuanian Cancer Registry. The data available for this analysis included patients’ sex, age, date of diagnosis, date of death, underlying cause of death, cancer location, and tumor stage. Patients were identified using the International Classification of Diseases (ICD-10) for diagnosis of primary colorectal cancer. Standardized mortality ratios (SMRs) were examined in relation to a number of factors: sex (separately for men and women), age interval at diagnosis (<50, 50–59, 60–69, 70–79, ≥80), time interval of diagnosis (1998–2002, 2003–2007, 2008–2012), time since colorectal cancer diagnosis (1–3, 4–6, 7–12 months, and 2–4 or >5 years), extent of disease, and cancer site (codes: C18–19, C20–21).

Study 7 [35] used the U.S. Surveillance, Epidemiology, and End Results database, and included patients diagnosed with rectal cancer (*n* = 187,996) and patients diagnosed with colon cancer (*n* = 443,368). The program provided data on cancer incidence, mortality, demographics, and survival. The outcome of the study was the cause of death reported as being self-inflicted and/or suicide (code 50220). The following factors were considered: age when diagnosis was made, sex, race (white, African American, or other), marital status (single, married, or unknown status), location of primary tumor (colon or rectum), stage of cancer when diagnosis was made (local or regional vs. distant), number of primary tumors, and whether surgery was performed at the primary site.

Study 8 [36] included 65,535 patients with a follow-up of 109,597 person-years. The SEER 18 registry database was queried for patients diagnosed between 1 January 1998 and 31 December 2011 who were diagnosed with gastric cancer, having one of the following histologic types: “carcinoma, not otherwise specified (NOS)”, “adenocarcinoma NOS”, “adenocarcinoma, intestinal type”, “carcinoma, diffuse type”, “tubular adenocarcinoma”, “adenocarcinoma with mixed subtypes”, “papillary adenocarcinoma, NOS”, “mixed cell adenocarcinoma”, “mucinous adenocarcinoma”, “mucin-producing adenocarcinoma”, or “signet-ring cell carcinoma”. Patients were excluded if the initial diagnosis was made at autopsy, if they were identified only by death certificate, or if survival was unknown.

Study no. 9 [37] identified 36,221 patients with adenocarcinoma of the pancreas, 30 of whom died by suicide during 22,248 person-years of follow-up in the SEER program of the National Cancer Institute. All patients classified with ICD-3 code 8140/3, adenocarcinoma not otherwise specified (NOS), which refers to adenocarcinoma of the pancreas, and excludes other malignant neoplasms such as neuroendocrine malignancies and premalignant neoplasms, were included in the study. Patients with other causes of death, including “accidents and adverse effects (50,210)”, “homicide and legal intervention (50,230)”, and “other cause of death (50,300)”, were not classified as suicides.

Study 10 [38] analyzed 76,955 patients with colorectal cancer from the Colorectal Cancer Database Sweden registry, including people diagnosed with rectal cancer between 1997 and 2016 and/or colon cancer between 2007 and 2016. The aim of the study was to compare the incidence of suicide in colorectal cancer patients with the incidence in a corresponding control cohort. Further objectives were to assess the risk of suicide within subgroups based on tumor location, gender, and surgical status. All included patients had a colorectal cancer diagnosis recorded in the Swedish Colorectal Cancer Register (SCRCR) and linked to national health and statistics registers.

## 4. Discussion

### 4.1. Is Suicide a Failure of Mental Adaptation to Cancer?

Mental adaptation to cancer involves complex processes, including accepting the diagnosis, adapting to physical limitations, and reorganizing life goals and priorities [39,40]. This adaptation is shaped by a complex interplay of emotional, physical, social, existential, and practical challenges. Each of these factors—emotional distress and depression, physical suffering, existential and spiritual distress, social and practical stressors, financial burden, and employment-related issues—compounds the psychological challenges that cancer patients face.

### 4.2. Emotional Distress and Depression

Emotional distress, particularly depression, is a significant factor in cancer patients’ ability to adapt mentally to their diagnosis [41]. Depression can magnify feelings of despair, making suicide seem like a way to escape unbearable emotional pain [42]. Moreover, depression often exacerbates other aspects of mental adaptation, including coping and resilience, further increasing suicide risk [43].

### 4.3. Physical Suffering and Symptom Burden

The severity of physical suffering may serve as an impediment to mental adaptation to cancer [44]. For numerous patients, the persistent nature of their pain or the incapacity to engage in typical daily activities can diminish their quality of life and self-esteem, potentially resulting in suicidal ideation [45].

### 4.4. Existential and Spiritual Distress

Many patients experience a spiritual crisis, especially when facing the possibility of an early death [46]. In our view, a failure in mental adaptation can, in this case, be linked to an inability to find spiritual or existential meaning in the face of cancer, which can elevate suicide risk.

#### 4.4.1. Social and Practical Stressors

Social isolation or loneliness is a significant risk factor for depression and suicide in cancer patients [46,47]. The physical limitations imposed by cancer treatment can make it difficult for patients to engage in social activities or maintain relationships. As a result, some patients may withdraw from friends, family, and social networks, leading to feelings of loneliness and abandonment. The loss of social connection can intensify feelings of helplessness, particularly if the patient feels that they can no longer contribute to their social environment or are a burden to others. Additionally, the stigma associated with certain cancers, such as those linked to smoking or alcohol use, can lead to social marginalization and further isolation [48]. Patients may avoid seeking support due to fear of being judged or misunderstood, deepening their sense of loneliness and despair [49]. Social and psychological factors, such as concerns about a loss of dignity, fear of being a burden to others, a loss of hope, and depression, were four of the most frequently cited reasons for requests for euthanasia [39].

#### 4.4.2. Financial Burden

One of the most significant practical stressors for cancer patients is the financial burden associated with treatment [50,51,52]. The stress of managing medical bills and the fear of losing financial stability can lead to feelings of helplessness, depression, and hopelessness about the future, which can negatively impact mental adaptation to cancer. This sense of financial crisis is a significant challenge to mental adaptation, and can amplify suicidal thoughts when patients see no way out of their financial and emotional turmoil.

#### 4.4.3. Employment and Career

The stigma associated with a cancer diagnosis may affect a patient’s ability to maintain professional relationships or return to work after treatment [53]. These challenges can intensify the emotional distress that patients experience, making it more difficult for them to mentally adapt to the realities of their illness. Returning to work after treatment can be difficult, and some individuals may feel that they are no longer capable of performing at the same level, leading to feelings of inadequacy. This can contribute to emotional distress, particularly if patients feel that they have lost their professional identity or fear they will not be able to return to their previous career.

In summary, each of these factors not only disrupts the mental adaptation process by creating psychological, social, and practical challenges, but also compounds the risk of suicidal ideation and, ultimately, suicide. The inability to adjust mentally, socially, and financially to the realities of cancer can push some patients to see suicide as a way to escape their overwhelming distress. The question of whether suicide indicates a failure of mental adaptation to cancer is complex. SEER databases lack detailed psychiatric data, focusing mainly on demographics and clinical information. Therefore, discussions on cancer and suicide, particularly regarding mental adaptation, are limited. This absence of data prevents a direct analysis of psychological factors influencing suicide risk in cancer patients.

#### 4.4.4. Sociodemographic and Clinical Factors Related to Suicide

The risk of suicide in cancer patients is shaped by both sociodemographic and clinical factors. Our review of 10 studies (involving 1,116,732 patients and 2711 suicides) highlights the complex interplay between these factors. Sociodemographic risk factors such as male gender, older age, unmarried status, and white ethnicity are prominent contributors to increased suicide risk. Additionally, clinical factors like advanced cancer stages, metastatic disease, and the type of treatment received (including surgery, chemotherapy, and radiotherapy) are also significant in influencing suicide risk.

Specifically, cancer patients in the advanced stages of disease or with metastatic cancer face the highest suicide risks. Moreover, those who receive no treatment or experience significant complications from treatments may also have higher suicide rates.

### 4.5. Gender Differences in Suicide Risk

Men tend to show higher suicide rates compared to women [29,30,31,32,33,35,36,37,38], a pattern that persists across many chronic illnesses, including cancer. This can be attributed to societal pressure to conform to traditional masculine ideals. These norms often discourage men from expressing vulnerability or seeking help for mental health issues, which exacerbates their distress. Additionally, men may be less likely to engage with social support networks, which are critical for managing emotional and psychological strain. In contrast, women face lower suicide risks and possess better coping mechanisms, including emotional expression and social support. In fact, while the suicide rate among women with cancer is lower, it remains elevated compared to that in the general population. However, most studies exclude LGBT populations, limiting the generalizability of the findings.

### 4.6. Marital Status and Social Support

A stable relationship or marriage as a sign of social support lowers the risk of suicide in the general population by reducing the risk of physical and psychiatric disorders [54,55,56] and increasing well-being and financial satisfaction [55]. The increased risk of suicide in unmarried men may be attributed to several factors, including social isolation, a lack of emotional support, and difficulty coping with the emotional and psychological burden of cancer. In addition, social and psychological factors (e.g., concerns about loss of dignity, fear of being a burden to others, a loss of a sense of dignity and hope, and depression) were four of the five most frequently cited reasons for euthanasia requests [39].

### 4.7. Geriatric Psycho-Oncology

Our analyses show that suicide risk increases significantly in those aged ≥60 years [30,31,32,33,34,37]. Older adults with cancer, especially those aged 65 and older, face challenges like comorbidities, frailty, cognitive issues, and emotional strain that impact their treatment and quality of life. Depression in older adults correlates with higher postoperative morbidity, disability, mortality, and greater resource needs, highlighting the importance of monitoring and intervention for this vulnerable group. Psychological interventions, such as counseling and therapy, are vital for managing emotional burdens, while collaboration among oncologists, geriatricians, psychologists, and social workers addresses the complex needs of elderly patients, enhancing treatment outcomes and quality of life.

### 4.8. Esophageal Cancer

Esophageal cancer patients exhibit high suicide rates in the first 5 years after diagnosis, with a notably high SMR in the first 2 months [31].

A study by Henson et al. [32] found that the suicide risk was highest within the first six months after diagnosis, but remained elevated for up to three years. The authors [32] highlight in their paper two key findings: first, that women had a threefold increased risk of suicide, independent of clinical depression; and second, that these findings suggest the need for better psychological support and suicidality screening for all cancer patients, particularly those at higher risk, immediately after diagnosis.

### 4.9. Hepatocellular Carcinoma

While pancreatic cancer also presents a higher risk of suicide, liver cancer exhibits a more pronounced immediate risk following diagnosis (particularly for those surviving for less than 2 months), whereas pancreatic cancer has a greater overall SMR [31]. The study of Chen et al. [30] found that while certain high-risk factors, such as an advanced disease stage or psychiatric illness, were associated with an increased risk of suicide, factors typically associated with cancer prognosis, such as marital status, histological grade, surgery, radiotherapy, and chemotherapy, did not show a strong correlation with suicide risk in HCC patients. The study highlights that the suicide rate in HCC patients was relatively low in the period between 2003 and 2016, which the authors attribute to the emergence of new and effective therapies. In addition to improving quality of life, these advances may have contributed to a better overall outcome for patients and reduced psychological distress, and thus a reduced risk of suicide.

### 4.10. Pancreatic Cancer

Patients with pancreatic cancer, similarly to those with esophageal and gastric cancers, face a significant suicide rate shortly after their diagnosis. Nonetheless, pancreatic cancer continues to be a high-risk cancer even beyond the initial months, as indicated by its overall standardized mortality ratio (SMR) of 6.43. Ma et al. [29] found that pancreatic neuroendocrine tumor (pNET) was associated with a lower suicide risk, suggesting that patients with pNET may experience a lessened psychological burden or better prognosis, leading to a reduced suicide risk. Ma et al. [29] recommend screening and subsequent suicide prevention in patients with pancreatic cancer who have unfavorable risk factors, such as being unmarried, white, or male. The fact that chemotherapy and surgical procedures appear to act as protective factors highlights the role of treatment optimism and medical intervention in reducing psychological distress [29].

According to Turaga et al. [37], suicide in patients aged 65 to 74 years who underwent surgery for pancreatic cancer was more likely to occur within the first 2 months after surgery. This is an important finding, but unsurprisingly, the reasons behind it are complex. This trend should encourage clinicians to perform effective screening and interventions in patients with this type of cancer, particularly during the first postoperative examination. 

### 4.11. Gastric Cancer

In 2016, Sugawara et al. [36] demonstrated that radiotherapy and surgery are protective factors against suicide in people with gastric cancer, and that people with cancer who did not receive or refused recommended treatment were at higher risk of suicide than people who received definitive treatment. A plausible explanation for the association between radiotherapy and lower suicide risk is that radiotherapy reduces tumor size, increases the resectability rate of surgery, improves prognosis, increases confidence in rehabilitation, and thereby alleviates patients’ pessimistic mood. In this study, we found that patients who did not receive or refused the recommended treatment had a higher risk of suicide, probably because patients in the advanced stages of the disease usually suffer from major psychological problems, including depression and anxiety.

### 4.12. Colorectal Cancer

Psychosocial status is critical, because colon cancer usually has a better prognosis and is associated with lower morbidity than cancers at other sites (e.g., stomach, lung, and liver). Construction of a temporary or definitive stoma is challenging because of physiological and social issues [57,58,59,60,61,62,63,64]. Psychosomatic care of patients after surgical treatment must include quality-of-life analysis with colostomy.

Sugawara et al. [36] and Larsson et al. [38] emphasize that rectal cancer is associated with high morbidity due to increased stomatal rates and the devastating effects of locally advanced and recurrent disease, including significant pain and spinal cord compression.

## 5. Conclusions

Patients who have been diagnosed with cancer have a significantly higher risk of suicide than patients in the general population. The risk is higher in men than in women, and is highest in people over 60. The risk of suicide is inversely related to the time since cancer diagnosis, and is highest within the first three to six months after diagnosis. In men, it is highest for cancers of the esophagus, stomach, and pancreas, and in women, it is highest in those with tumors of the colorectum.

Our comprehensive analyses of the impact of cancer diagnosis on suicide risk highlight the complex interplay of factors that contribute to suicide in cancer patients, including both physical and interpersonal elements.

In addition, recognition of the prevalent risk factors for suicide should include targeted intervention strategies and treatment plans that may reduce suicidality, such as aggressive treatment of pain, physical symptoms, delirium, and cognitive impairment. The aftermath of a patient’s suicide can be a challenging and stressful experience that can linger for healthcare providers, potentially leading to burnout and compromised patient care.

After a complete suicide, comprehensive mental healthcare should extend beyond the patient. The goal is to care for survivors, caregivers, and healthcare providers, destigmatize the tragedy of suicide, support the recovery process, and prevent further suicides by providing support services and psychosocial support to survivors. Good clinical practice also includes addressing the needs of affected family members to reduce the likelihood of complicated grief when a patient chooses to end their own life.

Finally, systematic changes in cancer treatment, such as frequent screening, increased surveillance, or active intervention in high-risk patients, can help to prevent unnecessary death.

### 5.1. Limitations

The present study is subject to several undeniable limitations, such as the amount of retrospective data available from the Surveillance, Epidemiology, and End Results Program (SEER), which makes it difficult to explain some of the results.

First, the SEER program does not have the necessary data sources to conduct further research on underlying variables such as comorbidities, cancer recurrence, socioeconomic status, health insurance, underlying psychiatric problems, suicide attempts, and information on treatment interventions. Second, but independently of SEER, seven of the nine included studies were conducted on members of the U.S. population, leaving no room for comparison of results with other countries with a high cancer burden. Third, the current coronavirus disease 2019 (COVID-19) pandemic has revealed glaring problems in the global healthcare system, and has affected the care of cancer patients. Unfortunately, however, reports on the prevalence and risk factors of suicidal ideation and suicide attempts among cancer patients during the COVID-19 pandemic are lacking.

### 5.2. Implications

This review highlights the importance of mental health professionals and clinicians more closely examining patients who are experiencing psychopathological issues and have cancer. It is important to ask these patients about their circle of support and their ability to request help from their environment. When distress is present, it is critical to assess the patient’s decision-making flexibility, consider the potential consequences of his/her actions (such as suicide), and adjust his/her behavior as appropriate.

### 5.3. Future Directions

#### 5.3.1. Clinical Practice Recommendations

Although suicide among cancer patients is rare, it remains a critical issue for patient care and requires effective management strategies. Identifying patients at increased risk of suicide is essential and should involve a comprehensive, multidisciplinary approach. This includes regular psychological assessments to monitor mental health symptoms, as well as creating robust support systems for patients. Healthcare providers should encourage open communication about emotional struggles and offer tailored therapeutic interventions to meet individual needs. Early intervention is key to reducing the risk of suicide, and providing patients with immediate emotional support can significantly enhance their quality of life. The goal is to ensure that patients receive the right care at the right time, addressing both their physical and emotional health, which ultimately contributes to better overall well-being and decreases the likelihood of suicide.

#### 5.3.2. Research Prospects

While suicide in cancer patients is a known issue, there is still a lack of comprehensive data, especially regarding how suicide rates vary over time in different countries. Much of the existing research is limited, and data from various parts of the world, especially low-resource countries, are sparse. To fill these gaps, future research should focus on a global perspective, comparing suicide rates among patients with digestive cancers and other cancer types. Researchers should also explore additional risk factors that may contribute to suicide, such as socioeconomic status, support systems, and treatment-related experiences. Furthermore, studies on younger cancer patients, especially teenagers and young adults, are limited. Our study had a lower age limit of 18 years, which prevented us from fully investigating the suicide risk in this group. Pooling data from different regions and utilizing advanced research methods, like the self-controlled case series approach, may help to better pinpoint when the suicide risk is highest, offering more insights into prevention. The development of innovative, technology-based solutions for suicide prevention in cancer patients is another promising research avenue. Online platforms, mobile applications, and wearable devices have the potential to provide real-time, accessible mental health support. Further studies should explore how these technologies can be integrated into cancer care to improve emotional well-being, offer timely interventions, and help to identify signs of suicidal behavior before the situation becomes critical. Evaluating the effectiveness of these tools in real-world settings will be key to their successful implementation in clinical practice. By focusing on both clinical improvements and research advancements, we can create a more comprehensive understanding of suicide risk in cancer patients and develop strategies to reduce these tragic outcomes.

## Figures and Tables

**Table 1:** Databases and search strategies used, and results obtained for each.

Database	Search Strategies
PubMed	Query box: (“suicide” [Mesh] AND (“digestive cancer” [Mesh] OR “esophageal cancer” [Mesh] OR “gastric cancer” [Mesh] OR “pancreatic cancer” [Mesh] OR “colorectal cancer”). In Embase, we used the search term (exp suicide) AND (digestive cancer system OR esophageal cancer OR gastric cancer OR pancreatic cancer OR colorectal cancer OR liver cancer) Refined by the following: Language: English; 2011–2024
PsycINFO	(exp suicide/OR “death” and “dying”) AND (exp “digestive cancer tract”/OR “esophageal cancer”/OR “gastric cancer”/OR “pancreatic cancer”/OR “colorectal cancer” OR “liver cancer”) Refined by the following: Language: English; 2011–2024
EMBASE	(exp suicide) AND (digestive cancer system OR esophageal cancer OR gastric cancer OR pancreatic cancer OR colorectal cancer OR “liver cancer”) Refined by the following: Language: English; 2011–2024
CINAHL	((MH “suicide”) OR (MM “death and dying”)) AND ((MH “digestive cancer system”) OR (MM “esophageal cancer”) OR (MM “gastric cancer” OR “pancreas cancer”/OR “colorectal cancer” OR “liver cancer”)) Refined by the following: Language: English; 2011–2024
Web of Science	(TOPIC: (“suicide”) OR Title: (“death” and “dying”) AND (TOPIC: (“mortality”) OR TOPIC: (“digestive system cancer”) OR TOPIC: (“survival rate”) OR TOPIC: (“premature mortality”) OR TOPIC (“esophageal cancer”) OR TOPIC (“gastric cancer”) OR TOPIC (“pancreas cancer”)/OR TOPIC (“colorectal cancer”) OR TOPIC (“liver cancer”) Refined by the following: Language: English; 2011–2024

**Table 2:** Characteristics of the included studies describing suicide in patients with cancer of the digestive tract.

Author/Year/	Country	General Study Aim	No. of Suicides/No. of Patients	Time Period	Cancer Localization	Sociodemographic Risk Factors	Psychological Factors	Clinical Factors	Factors Related to Suicide	SMR (Standardized Mortality Ratio)	Link
Ma et al. 2022 [29]	USA	To determine the standardized mortality ratio (SMR) of suicide and risk factors associated with suicide in patients with pancreatic cancer, compared with the general population, to provide evidence for prevention.	180/199,604	2000–2018	Pancreatic cancer	Male gender, unmarried, white ethnicity	-	Pancreatic neuroendocrine tumors (pNETs) have a lower suicide risk.	PDAC patients have a higher risk of suicide. No treatment (radiotherapy, chemotherapy, surgery) increases suicide risk.	SMR: 7.06 for males	https://www.ncbi.nlm.nih.gov/pmc/articles/PMC9437642/ (accessed on 2 August 2024),
Chen et al. 2021a [30]	USA	To determine the suicide rate and SMRs in patients with liver cancer, in comparison to the U.S. general population, and to identify any relevant risk factors for suicide, using data from the SEER database.	70/102,567	1975–2016	Hepatocellular cancer	Male gender, older individuals (63–105 years), unmarried, white individuals	-	Higher suicide rates in patients with shorter survival, especially <2 months. Advanced cancer stage associated with higher suicide rates.	Higher suicide rates in the first five years after diagnosis: <2 months SMR = 26.78.	SMR = 26.78 for <2 months	https://www.ncbi.nlm.nih.gov/pmc/articles/PMC7538389/ (accessed on 25 August 2024)
Chen et al. 2021 b [31]	USA	To evaluate suicide rates and SMRs relative to the U.S. general population and identify underlying causes of suicide using the SEER database.	161/69,773	1975–2016	Esophageal cancer	Male gender, older age, unmarried, white ethnicity	-	Grade III tumors associated with higher suicide rates. No surgery or chemotherapy increases suicide rates.	Patients with esophageal cancer had significantly higher suicide rates, particularly within the first five years after diagnosis: <2 months: SMR = 216.79 2–11 months: SMR = 21.57 12–59 months: SMR = 3.89	SMR = 216.79 for <2 months	https://pubmed.ncbi.nlm.nih.gov/34548616/ (accessed on 28 August 2024)
Henson et al. 2019 [32]	England	To calculate the suicide risk in cancer patients in England, and to identify potential risk factors involved in needs-based psychological assessment.	Liver cancer: 10/51,800, pancreatic cancer: 33/121,207, esophageal cancer: 57/122,132, stomach cancer: 59/128,965, colorectal cancer: 349/578,270	1995–2015	Esophagus, stomach, pancreatic, liver, and colorectal cancers	Male gender, white ethnicity cancer patients aged 60+	-	Pancreatic cancer has a 3.89-fold increase in suicide risk.	Suicide risk is highest in the first 6 months after diagnosis. The risk rises for the first 3 years, and declines thereafter.	SMR for pancreatic cancer = 3.89	https://www.ncbi.nlm.nih.gov/pmc/articles/PMC6583458/ (accessed on 2 September 2014)
Anderson et al. 2018 [33]	USA	To investigate the suicide rate in various subgroups, such as the site of cancer, stage of cancer, time since diagnosis, and various patient characteristics.	Esophageal cancer: 88/43,912, colon cancer: 311/318,861, rectum and rectosigmoid junction: 185/134,913, liver and intrahepatic bile duct: 47/81,684, pancreas: 93/119,194, stomach: 81/76,313, other digestive organs: 76/81,415	2000–2014	Esophageal, pancreatic, gastric, rectum and rectosigmoid junction, pancreas, liver, and intrahepatic bile duct cancers	Older age (80+ years), male gender, white ethnicity cancer patients, unmarried	-	Pancreatic cancer and esophageal cancer show the highest suicide risk.	Highest suicide risk in the first 5 years of diagnosis for all cancers. Metastatic-stage cancer patients have the highest SMR (4.60).	SMR for pancreatic cancer = 5.28	https://pubmed.ncbi.nlm.nih.gov/29956393/ (accessed on 18 September 2024)
Dulskas et al. 2018 [34]	Lithuania	To estimate the suicide risk in patients diagnosed with colorectal cancer in Lithuania.	67/19, 409	1998–2012	Colorectal cancer	Female gender, aged 60+	-	Stage IV cancer patients have a fourfold increased risk of suicide.	Suicide risk is highest in the first 3 months after cancer diagnosis (SMR = 4.00).	SMR = 4.00 for Stage IV	https://pubmed.ncbi.nlm.nih.gov/30617411/ (accessed on 8 October 2024)
Samawi et al. 2017 [35]	USA	To examine the incidence and predictors of suicide in patients diagnosed with a form of colorectal cancer (colon cancer or rectal cancer).	Rectal cancer: 337/187,996, colon cancer: 611/443,368	1973–2009	Colorectal cancer	Male, white race (was a predictor of suicide in the colon cancer cohort) Older age was an independent predictor of suicide in both colon cancer and rectal cancer	-	Metastatic disease and lack of primary resection predict suicide risk.	Suicide risk is higher in individuals with older age, male sex, white race, and metastatic disease.	SMR = 2.3 for metastatic disease	https://www.ncbi.nlm.nih.gov/pmc/articles/PMC5736490/ (accessed on 24 October 2024)
Sugawara et al. 2016 [36]	USA	To investigate suicide rates in patients diagnosed with gastric cancer and to identify relevant factors that can increase the suicide risk.	68/65,535	1998–2011	Gastric cancer	Male gender, older age, white race, unmarried individuals	-	Advanced cancer (Stage IV) and metastatic disease significantly increase suicide risk.	Suicide risk is highest within the first 3–6 months after diagnosis. Non-treatment (surgery, chemotherapy, radiotherapy) predicts a higher suicide risk.	White race (SMR = 3.23) Unmarried status (SMR = 2.01) Distant-stage disease (SMR = 2.90).	https://pubmed.ncbi.nlm.nih.gov/27307574/ (accessed on 3 November 2024)
Turaga et al. 2011 [37]	USA	To determine suicide rates in patients diagnosed with pancreatic cancer using population-based data, which were used to identify patient, disease, and treatment characteristics associated with suicide.	30/36,221	1995–2005	Pancreatic cancer	Male gender, age ≥ 60 years, unmarried		Surgery is associated with increased suicide risk, especially postoperatively.	Suicide risk in male pancreatic cancer patients is nearly 11 times higher than the general population. Surgery increases suicide risk in the early postoperative period.	SMR for males = 11.00	https://pubmed.ncbi.nlm.nih.gov/20824626/ (accessed on 8 November 2024)
Larsson et al. 2024 [38]	Sweden	To investigate the prevalence of suicide in a national cohort of patients with newly diagnosed colorectal cancer compared with a matched control group, to determine whether patients with colorectal cancer have an increased incidence of suicide.	24/64,866	1997–2006 (colorectal cancer) 2008–2017 (colon cancer)	Colorectal cancer	Male gender, unmarried patients	-	Surgery significantly increases suicide risk. Non-surgical patients have a higher suicide risk.	Suicide risk is highest in the first year following diagnosis (SMR = 1.86). Non-operated patients have a higher suicide risk (SMR = 7.03).	SMR for non-operated patients = 7.03	https://pubmed.ncbi.nlm.nih.gov/38831481/ (accessed on 24 December 2024)

## Data Availability

Not applicable.
